# *Notes from the Field:* Locally Acquired Mosquito-Transmitted (Autochthonous) *Plasmodium falciparum* Malaria — National Capital Region, Maryland, August 2023

**DOI:** 10.15585/mmwr.mm7241a3

**Published:** 2023-10-13

**Authors:** Monique Duwell, Timothy DeVita, David Torpey, Jenny Chen, Robert A. Myers, Kimberly Mace, Alison D. Ridpath, Wycliffe Odongo, Brian H. Raphael, Audrey Lenhart, Jon Eric Tongren, Stephen Stanley, David Blythe

**Affiliations:** ^1^Infectious Disease and Epidemiology and Outbreak Response Bureau, Maryland Department of Health; ^2^Division of Parasitic Diseases and Malaria, Global Health Center, CDC; ^3^Maryland Department of Health Laboratories Administration.

Although malaria was eliminated in the United States in the mid-1950s, approximately 2,000 malaria cases are imported into the United States from regions with endemic disease transmission each year, including approximately 200 in Maryland* (Figure) (*1*). *Anopheles* mosquito species that can transmit malaria exist in many areas in the United States (*2*). Locally acquired mosquito-transmitted (i.e., autochthonous) cases have not been identified since 2003; however, these imported cases represent a potential source of infection. In mid-2023, eight autochthonous malaria cases (*Plasmodium vivax*) were identified in Florida and Texas (*3*); in both states, the autochthonous cases occurred in the vicinity of an imported malaria case.

**FIGURE:**
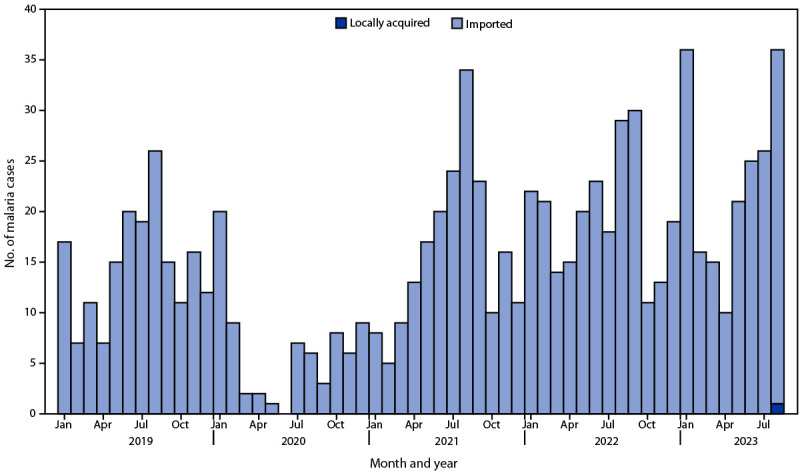
Malaria cases, by month and year* — Maryland, January 1, 2019–August 31, 2023^†^^,^^§^ * Based on symptom onset date or diagnosis date, if onset date is unknown. ^†^ Data from 2023 are preliminary. ^§^ Cases of imported malaria are influenced by travel, and high numbers of cases occur during July–September and in January. The COVID-19 pandemic affected the numbers of cases imported into the United States.

## Investigation and Outcomes

On August 6, a previously healthy resident of the Maryland National Capital Region was evaluated for a 7-day history of fever, malaise, and myalgias. In the months preceding symptom onset, the patient reported daily walks near home and an occurrence of a tick attachment but no international travel, blood transfusions, intravenous drug use, or other potential exposures to bloodborne pathogens.

Initial hospital laboratory testing revealed anemia, thrombocytopenia, hyperbilirubinemia, and intraerythrocytic parasites that raised concern for babesiosis or malaria. The patient was admitted to the hospital and, given the absence of international travel and the reported tick exposure, empiric treatment for presumed babesiosis^†^ was initiated. On August 9, a thin blood smear obtained at the time of admission was reported to show *Plasmodium falciparum* malaria with 3.2% parasitemia. Blood smear telediagnosis at CDC could not conclusively differentiate between malaria and babesia parasites from the images provided. In accordance with Maryland law, the smear and whole blood specimen were also submitted to the Maryland Department of Health (MDH) public health laboratory. Because the patient had no reported international travel and did have a history of tick exposure, as well as documented clinical improvement (reduction in parasitemia to 0.2%), the patient was discharged on August 10 with instructions to complete a 7-day babesiosis treatment course.^§^

On August 15, testing at MDH public health laboratory identified *P. falciparum* using smear microscopy, the BinaxNOW Malaria rapid diagnostic test (Abbott), and 18S rRNA polymerase chain reaction (PCR)*.* On August 18, CDC confirmed *P. falciparum* infection by 18S rRNA PCR; the *Babesia spp.* PCR test result was negative. Considering these findings, after completion of the babesiosis treatment, the patient received a course of artemether-lumefantrine.

MDH and the local health department first confirmed that all household members were asymptomatic and that the patient had not traveled internationally recently. Next, a public notice was issued, urging residents to avoid mosquitoes and to seek medical attention for malaria symptoms; Maryland clinicians and public health professionals were alerted to the case and provided recommendations to prioritize timely diagnosis, treatment, and public health reporting. To identify other potential malaria cases in local hospitals, active case finding was implemented. In coordination with the Maryland Department of Agriculture, mosquito surveillance was conducted by trapping *Anopheles* mosquitoes^¶^ and applying multiple rounds of larvicide and adulticide. No geographically proximate malaria cases (i.e., within <5 miles [<8 kms] of the patient’s residence) during the preceding month were identified, and although *Anopheles* mosquitoes were present near the patient’s home, none of the 21 *Anopheles* mosquitoes tested at CDC was positive for *P. falciparum*. The source of the patient’s exposure remains unknown. To date, no additional autochthonous malaria cases of any parasite species have been identified in Maryland. This activity was reviewed by CDC, deemed not research, and was conducted consistent with applicable federal law and CDC policy.**

## Preliminary Conclusions and Actions

This case underscores common challenges in malaria diagnosis, including differentiation from babesiosis, and potential for sporadic autochthonous malaria cases in the United States and highlights the need for coordinated efforts among public health officials, clinicians, laboratories, and the public to prevent, detect, and respond to such cases. Proposed interventions include ensuring that travelers to regions where malaria is endemic take appropriate malaria chemoprophylaxis to reduce both personal and community risk. Improving capacity for timely malaria diagnosis through blood smear examination, rapid diagnostic test availability and use, PCR for species confirmation,^††^ and early request for CDC support are also recommended.^§§^

## Footnotes

*
https://health.maryland.gov/phpa/OIDEOR/CIDSOR/Pages/disease-conditions-count-rates.aspx

† Atovaquone (750 mg twice daily), azithromycin (500 mg twice daily), and doxycycline (100 mg twice daily) for 7 days.

§ Atovoquone is a component of Malarone (atovoquone and proguanil), which is used for *P. falciparum* prophylaxis and treatment, and doxycycline is used for *P. falciparum* prophylaxis.

¶ *Anopheles quadrimaculatus, A. punctipennis, *and* A. crucians/bradleyi*.

** 45 C.F.R. part 46.102(l)(2), 21 C.F.R. part 56; 42 U.S.C. Sect. 241(d); 5 U.S.C. Sect. 552a; 44 U.S.C. Sect. 3501 et seq.

†† The Council of State and Territorial Epidemiologists recommends malaria PCR testing to confirm *Plasmodium* and species.

§§ CDC provides clinical assistance through the Malaria Hotline at 770-488-7788 or 855-856-4713 (toll-free) Monday–Friday, 9 a.m.–5 p.m. EST. After hours, on weekends, and on federal holidays, health care providers can call 770-488-7100 and ask to speak with the malaria clinician on call and diagnostic laboratory support.
